# Permanent magnet synchronous motor demagnetization fault diagnosis based on PCA-ISSA-PNN

**DOI:** 10.1038/s41598-024-72596-5

**Published:** 2024-09-20

**Authors:** Yinquan Yu, Yang Li, Dequan Zeng, Yiming Hu, Jinwen Yang

**Affiliations:** 1 Institute of Precision Machining and Intelligent Equipment Manufacturing, Key Laboratory of Conveyance and Equipment of Ministry of Education, East China Jiaotong University, Nanchang, 330013 China; 2 School of Mechatronics and Vehicle Engineering, East China Jiaotong University, Nanchang, 330013 China

**Keywords:** Permanent magnet synchronous motor, Principal component analysis algorithm, Improved sparrow search algorithm, Probabilistic neural network, Electrical and electronic engineering, Mechanical engineering

## Abstract

Aiming at the demagnetization fault problem of the permanent magnet synchronous motor (PMSM), a demagnetization fault diagnosis method based on the combination of the principal component analysis (PCA) algorithm, the improved sparrow search algorithm (ISSA), and the probabilistic neural network (PNN) algorithm is proposed. First, the principal components of phase currents are extracted using PCA. Second, ISSA is used to optimize the smoothing coefficients of the PNN algorithm, and the optimized PNN algorithm is combined with PCA to obtain the PCA-ISSA-PNN fault diagnosis model. Finally, the established fault diagnosis model was tested using the current data collected from the experiments and compared with the fault diagnosis indexes and optimization performance of the conventional PNN, PCA-PNN, PCA-GA (genetic algorithm)-PNN, PCA-DA (dragonfly algorithm)-PNN, PCA-GTO (artificial gorilla troop optimizer)-PNN, PCA-AHA-PNN, and PCA-SSA-PNN. The test results show that the fault diagnosis accuracy of PCA-ISSA-PNN reaches 95.83%, and the fault diagnosis indexes are significantly higher than those of PNN, PCA-PNN, PCA-GA-PNN, and PCA-DA-PNN; its optimization performance is also significantly better than that of PCA-GTO-PNN, PCA-AHA-PNN, and PCA-SSA-PNN, which verifies the accuracy and efficiency of the proposed method.

## Introduction

Permanent magnet synchronous motor (PMSM) is widely used in new energy vehicles, aerospace, transportation, and other fields because of its simple structure, high efficiency, high power density, and low noise^1–3^. However, due to the environment and temperature during operation, PMSM inevitably produces faults that affect the normal operation of the machine or even burn out, paralyzing the whole system and causing economic losses. Therefore, the study of PMSM fault diagnosis methods is of enormous significance for early detection of motor faults and saving maintenance costs.

Currently, there are numerous permanent magnet synchronous motor fault diagnosis methods, such as the fault diagnosis method based on the analytical model of the motor, the fault diagnosis method based on feature signal extraction, the motor fault diagnosis method based on artificial intelligence, and so on. The fault diagnosis method is based on the motor's analytical model^4^, which analyzes the fault using an accurate mathematical model. This method has the advantage of being able to diagnose a certain type of fault in the motor with high accuracy, but it is difficult to establish accurate mathematical models for different faults in different motors^5^. For example, S. Moon et al. proposed a method to diagnose demagnetization faults based on the structure of PMSM, addressing the challenge of accurately diagnosing the degree of demagnetization due to the change in inductance after motor demagnetization. They achieved this by calculating the inductance, taking into account the magnetic saturation effect, and combining it with the method of least squares. The experimental results demonstrate that this method can accurately track the change in inductance and accurately diagnose the degree of demagnetization of the permanent magnets^6^. A study by D.S.B. Fonseca et al. looked at how well the motor works when it's healthy and when it has a turn-to-turn short-circuit fault. They created a dynamic model of the motor's fault in MATLAB/Simulink and simulated and tested it with turn-to-turn short-circuit faults. The results show that the simulation results and experiment results match well, which shows that the proposed model can accurately simulate the PMSM fault state^7^. The fault diagnosis method relies on feature signal extraction^8^, utilizing signal processing to extract fault features from the voltage, current, and other running motor data. For instance, J. Gao et al. extracted the phase currents in the permanent magnet synchronous motor, converted the current signal from the time domain to the position domain with constant amplitude and frequency, and then used FFT analysis to diagnose demagnetization faults in both smooth and non-smooth conditions^9^. However, the process of converting the current signals proved to be complex and challenging to implement. J. Hang et al. utilized the wavelet transform to extract fault features from the cost functions found in PMSM model predictive control (MPC) systems. They then diagnosed turn-to-turn short-circuit faults by monitoring the normalized energy-dependent eigenvectors derived from the wavelet transform coefficients^10^. However, the selection of wavelet basis functions significantly influences the results, and determining these wavelet basis functions can be challenging. An artificial intelligence-based fault diagnosis method employs intelligent classification algorithms to pinpoint faults^11^. For example, C.-S. Wang et al. can achieve the detection of demagnetization faults, bearing faults, and rotor eccentricity faults in motors by analyzing the combined features of motor torque and current signals using a one-dimensional convolutional neural network^12^. The advantage of this method is that feature extraction can extract multi-scale features from complex conditions, but the amount of experimental data used is large, and when the amount of experimental data is small, overfitting phenomena occur, so it is not applicable to small samples. X. Song et al. used the S-transform to perform time–frequency decomposition of the back EMF signal of a permanent magnet linear synchronous motor in order to extract the feature parameters, establish the feature vectors by comparing the standard deviation values and similarity of different parameters, then use the particle swarm algorithm to optimize the regularization parameter and the width of the kernel function in the least-squares support vector machine, and finally use the optimized algorithm to perform the fault diagnosis, which can accurately identify the position of the demagnetization of the motor and the degree of demagnetization^13^, but the acquisition of the back EMF signals usually involves installing additional sensors, which increases the unnecessary cost.

The probabilistic neural network (PNN) algorithm is an efficient supervised pattern classification method that has the advantages of a simple network structure, fast computational speed, high computational accuracy, good classification effect, etc. It is particularly well-suited for fault diagnosis and classification problems with small samples, and it has a wide range of applications in the field of fault diagnosis^14^. X. Dai et al. used variational modal decomposition (VMD) on PMSM current signals to find the intrinsic modal component (IMF). They then used the energy value of the IMF as the eigenvector, the fuzzy C-mean algorithm to find the clustering centers of the fault data, and the PNN to sort the faults into two groups: turn-to-turn short-circuit faults and demagnetization faults^15^. In their study, J. Ding et al. used a variational particle swarm algorithm to find the best number of decompositions and penalty factors in the VMD. They then used the sample entropy of the IMF as the feature vector and combined it with PNN to correctly classify gear faults^16^. Nagi et al. extracted the color features of glucose leaves using fuzzy color histograms, and then used the PNN algorithm to accurately classify grapevine diseases, thereby preventing yield loss^17^. However, the above study's use of empirically set smoothing coefficients in the PNN significantly impacted the diagnostic accuracy.

Based on the above research, this paper proposes a PCA-ISSA-PNN-based demagnetization fault diagnosis method to improve the accuracy of PMSM fault diagnosis and reduce its cost. This method combines feature signal extraction and artificial intelligence, using the interior PMSM as the research object. The method first uses the principal component analysis algorithm to extract the principal components of the current signal, then uses ISSA to optimize the smoothing coefficients in the PNN, and combines the optimized PNN algorithm with PCA to get the fault diagnosis model of PCA-ISSA-PNN. Finally, the established fault diagnosis model was tested using the current data collected from the experiments and compared with the fault diagnosis indexes and optimization performance of the conventional PNN, PCA-PNN, PCA-GA (genetic algorithm)-PNN, PCA-DA (dragonfly algorithm)-PNN, PCA-GTO (artificial gorilla troop optimizer)-PNN, PCA-AHA-PNN, and PCA-SSA-PNN. The test results show that the fault diagnosis accuracy of PCA-ISSA-PNN reaches 95.83%, and the fault diagnosis indexes are significantly higher than those of PNN, PCA-PNN, PCA-GA-PNN, and PCA-DA-PNN; its optimization performance is also significantly better than that of PCA-GTO-PNN, PCA-AHA-PNN, and PCA-SSA-PNN, which verifies the accuracy and efficiency of the proposed method.

The method of this paper has the following advantages compared with previous research methods:

1. The PCA-ISSA-PNN method is a fault diagnosis method that combines feature signal extraction with artificial intelligence. There is no need to establish an accurate mathematical model.
2. The PCA extraction of fault signals method has simple steps, which can reduce the data dimension and improve operational efficiency without the need for complex data conversion.
3. The method uses a small number of samples, which can improve operational efficiency. The use of current signals for diagnosis does not require the installation of additional sensors, which reduces costs.
4. The use of ISSA to optimize the smoothing coefficient in the PNN improves diagnostic accuracy.

## PMSM mathematical model

The voltage equation of the PMSM on the d-q axis in the synchronous rotating coordinate system can be expressed^18^ as:

1 $$\begin{aligned}\left\{\begin{aligned} & {u}_{d}=R{i}_{d}+{L}_{d}\frac{{di}_{d}}{dt}-{\omega }_{e}{L}_{q}{i}_{q}\\ & {u}_{q}=R{i}_{q}+{L}_{q}\frac{{di}_{q}}{dt}-{\omega }_{e}{(L}_{q}{i}_{q}+\psi ) \end{aligned}\right.\end{aligned}$$

where ${u}_{d}$,${u}_{q}$,${i}_{d}$,${i}_{q}$ are the stator voltage and stator current of d-q, respectively. ${L}_{d}$,${L}_{q}$ are the stator inductance of the d-axis and q-axis, respectively, and *R* is the winding resistance. ${\omega }_{e}$ is the electrical angular velocity, and $\psi$ is the magnetic chain of the permanent magnet.

The electromagnetic torque equation can be expressed^18^ as:

2 $$\begin{array}{c}{T}_{e}=\frac{3}{2}{p}_{n}{i}_{q}\left[{i}_{d}\left({L}_{d}-{L}_{q}\right)+\psi \right]\end{array}$$

where ${T}_{e}$ is the electromagnetic torque and ${p}_{n}$ is the number of pole pairs.

When demagnetization of the permanent magnet occurs, the magnetic chain in the three-phase winding of the PMSM, which is generated by the no-load air-gap magnetism of the permanent magnet, can be expressed^15^ as:

3 $$\begin{aligned}{c}\left\{\begin{aligned}{c} & {\psi }_{A}=\sum_{k=1}^{\infty }{\psi }_{\frac{k}{{p}_{n}}}\text{cos}\left(\frac{k}{{p}_{n}}\theta \right)\\ & {\psi }_{B}=\sum_{k=1}^{\infty }{\psi }_{\frac{k}{{p}_{n}}}\text{cos}\left(\frac{k}{{p}_{n}}\left(\theta -\frac{2}{3}\pi \right)\right)\\ & {\psi }_{C}=\sum_{k=1}^{\infty }{\psi }_{\frac{k}{{p}_{n}}}\text{cos}\left(\frac{k}{{p}_{n}}\left(\theta +\frac{2}{3}\pi \right)\right)\end{aligned}\right.\end{aligned}$$

where ${\psi }_{A}$, ${\psi }_{B}$, ${\psi }_{C}$ are the magnetic chains of the three-phase windings, $k$ is a positive integer, and $\theta$ is the angle between the air-gap magnetic field axis of the permanent magnet and phase A of the motor.

The magnetic chain equation of the PMSM in the d-q axis can be expressed^15^ as:

4 $$\begin{aligned}  \left\{\begin{aligned}\begin{aligned} & {\psi }_{d}=\psi +{L}_{d}{i}_{d}\\ & {\psi }_{q}={{L}_{q}i}_{q} \end{aligned}\\ \end{aligned}\right.\end{aligned}$$

where ${\psi }_{d}$, ${\psi }_{q}$ are the magnetic chains of the d-axis and q-axis, respectively.

The above model shows that when the PMSM demagnetizes, the magnetic chain changes and affects the current, allowing us to select the current signal as the fault feature vector.

## Relevant theories

### Principal component analysis algorithm

Principal component analysis (PCA) is a technique commonly used to analyze large datasets and can be used to reduce high-dimensional data to the best feasible set of features^19–21^. PCA can significantly reduce the complexity of the neural network, thus improving the network recognition rate and prediction accuracy. Therefore, this paper uses PCA to extract data features to enhance fault diagnosis accuracy.

The specific steps of PCA are as follows:

1. Select test samples and training samples to build the initial feature matrix* X* as follows:
  $$X=\left(\begin{array}{ccc}{X}_{11}& \dots & {X}_{1m}\\ \dots & \dots & \dots \\ {X}_{n1}& \dots & {X}_{nm}\end{array}\right)$$
  where *n* is the number of samples and *m* is the initial feature dimension.
2. Calculate the correlation coefficient matrix;
3. Calculate the eigenvalues ${\lambda }_{i}(i=1,\cdots ,m)$ of correlation coefficient matrix and arrange ${\lambda }_{i}$ in ascending order. Calculate the accumulative contribution $\sum_{i=1}^{h}{\lambda }_{i}/\sum_{i=1}^{m}{\lambda }_{i}$, and determine the number of principal component dimensions *h*.
4. Calculate the principal components.

### Probabilistic neural network algorithm

Probabilistic neural network (PNN) is a feed-forward neural network model based on the Bayesian minimum risk criterion and the Parzen window function probability density estimation method proposed by Specht in 1990^14^, which has been widely used in different scientific fields^22^. The PNN method is swift and more accurate compared to other neural network methods^23^.

**Fig. 1:**
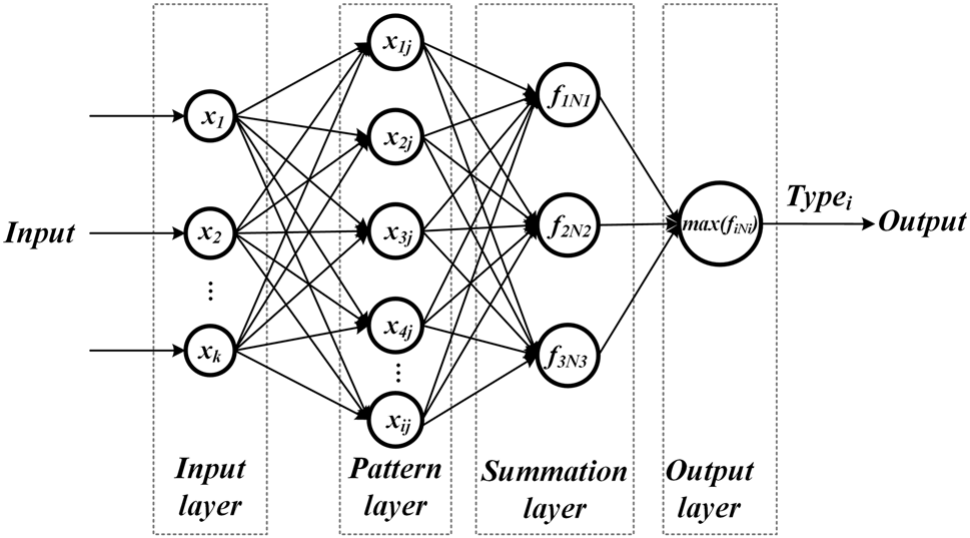
The structure of PNN.

A PNN network usually consists of four layers: the input layer, the pattern layer, the summation layer, and the output layer^24^. The structure of PNN network is shown in Fig. 1, assuming that the input test sample is $x={(x}_{1}{,x}_{2},\cdots ,{x}_{k})$, the training sample of pattern layer is ${x}_{ij}={(x}_{1j}{,x}_{2j},\cdots ,{x}_{ij})$, and the output is Type_i_.

Layer 1 of the PNN is the input layer, which receives the feature vectors of the test samples that need to be classified for fault classification and passes them to the network. Layer 2 is the pattern layer, which is used to compute the similarity between the input feature vectors and the patterns of each category in the training set. Its input–output relationship can be determined by Eq. (5):

5 $$\begin{array}{c}{\delta }_{ij}\left(x\right)=\frac{1}{{\left(2\pi \right)}^\frac{k}{2}{\sigma }^{k}}exp\left[-\frac{{\left(x-{x}_{ij}\right)}^{T}\left(x-{x}_{ij}\right)}{2{\sigma }^{2}}\right]\end{array}$$

where ${x}_{ij}$ is the feature vector of the jth sample of the ith class in the training sample, $x$ is the feature vector of the prediction sample, $k$ is the sample dimension, and $\sigma$ is the smoothing coefficient.

Layer 3 is the summation layer, which serves to compute the probability accumulation of a particular class to obtain the probability of belonging to a particular class of faults. The summation layer is calculated by Eq. (6), which sums up the mode layer units in each class.

6 $$\begin{array}{c}{f}_{i{N}_{i}}\left(x\right)=\frac{1}{{N}_{i}}\sum_{j=1}^{{N}_{i}}{\delta }_{ij}\left(x\right)\end{array}$$

where ${f}_{i{N}_{i}}\left(x\right)$ is the probability density function of *x* belonging to the ith class, and ${N}_{i}$ is the number of training samples in the ith class.

Layer 4 is the output layer, which serves to output the fault category of the motor.

Combining Eq. (5) with Eq. (6), the formula for the probability density is obtained as:

7 $$\begin{array}{c}{f}_{i{N}_{i}}\left(x\right)=\frac{1}{{\left(2\pi \right)}^\frac{k}{2}{\sigma }^{k}}\cdot \frac{1}{{N}_{i}}\cdot \sum_{j=1}^{{N}_{i}}\text{exp}\left[-\frac{{\left(x-{x}_{ij}\right)}^{T}\left(x-{x}_{ij}\right)}{2{\sigma }^{2}}\right]\end{array}$$

Usually, the smoothing coefficient $\sigma$ in PNN is set based on experience. If it is not set properly, it will affect the accuracy of recognition. Therefore, in order to improve recognition accuracy, this paper adopts the ISSA algorithm to optimize the smoothing coefficient $\sigma$ in PNN.

### Improved sparrow search algorithm

The sparrow search algorithm (SSA) is an intelligent optimization algorithm proposed in 2020 to mimic the predatory and anti-predatory behavior of sparrows^25^. During sparrow foraging, sparrows are categorized into discoverers and followers^26^. Discoverers are responsible for finding and guiding the way to food areas; they have high energy and provide foraging information to the entire population. Followers, on the other hand, are lower in energy and rely on the guidance of the finders for food^27,28^.

During each iteration, the position update formula of the discoverer can be described^29^ as:

8 $$\begin{array}{c}{X}_{i,j}^{t+1}=\left\{\begin{array}{c}{X}_{i,j}^{t}\cdot \text{exp}\left(-\frac{i}{\alpha \bullet {iter}_{max}}\right),{ \, \, if \, R}_{2}<ST \\ {X}_{i,j}^{t}+Q\cdot L , { \, \,\, \,\, \,\, \,\, \,\, \,\, \,\, \,\, \,\, \,\, \,\, \,\, \,\, \,if\, R}_{2}\ge ST \end{array}\right.\end{array}$$

where $t$ is the current iteration number, ${iter}_{max}$ is the maximum iteration number, and ${X}_{i,j}^{t+1}$ is the position information of the ith sparrow in the jth dimensional space .$\alpha$ is a uniform random number of $\left(\left.\text{0,1}\right]\right.$, ${R}_{2}$ is an alarm value of $\left.\left[\text{0,1}\right.\right]$, and $ST$ is a safety value of $\left.\left[\text{0.5,1}\right.\right]$. $Q$ is a random number and obeys a normal distribution, and $L$ is a $1\times \text{d}$ dimensional matrix with all 1 elements. When ${R}_{2}<ST$, it means that there is no predator around the population, and the discoverer can search widely for food. When ${R}_{2}\ge ST$, it means that a sparrow has discovered the predator and alarmed the police, then all sparrows have to fly quickly to a safe place to forage.

The follower's position update formula can be described as:

9 $$\begin{array}{c}{X}_{i,j}^{t+1}=\left\{\begin{array}{c}Q\cdot \text{exp}\left(\frac{{X}_{worst}^{t}-{X}_{i,j}^{t}}{{i}^{2}}\right), \, \, \, \,\, \,\,\, \,\, \, \, \,\, \,\, \,\, \,\, i>n/2 \\ {X}_{p}^{t+1}+\left|{X}_{i,j}^{t}-{X}_{p}^{t+1}\right|{A}^{+}\cdot L, \, \, \, i\le n/2\end{array}\right.\end{array}$$

where ${X}_{p}^{t+1}$ is the optimal position that the discoverer is currently positioned at, and ${X}_{worst}^{t}$ is the worst position that the discoverer is currently positioned at. $A$ is a $1\times \text{d}$ dimensional matrix with elements equal only to 1 or -1 and ${{A}^{+}={A}^{T}(A{A}^{T})}^{-1}$^26^ . When $i>n/2$, it means that the ith follower cannot grab food and has to go to other regions to feed. When $i\le n/2$, it means that the follower is feeding around the optimal individual ${X}_{p}$.

When there is a danger, the sparrow moves towards a safe area. The position update equation can be described as:

10 $$\begin{array}{c}{X}_{i,j}^{t+1}=\left\{\begin{array}{c}{X}_{best}^{t}+\beta \cdot \left|{X}_{i,j}^{t}-{X}_{best}^{t}\right|,{ \, f}_{i}>{f}_{g} \\ {X}_{i,j}^{t}+K\cdot \left[\frac{\left|{X}_{i,j}^{t}-{X}_{worst}^{t}\right|}{\left({f}_{i}-{f}_{w}\right)+\varepsilon }\right], { \,\, f}_{i}={f}_{g} \end{array}\right.\end{array}$$

where ${X}_{best}^{t}$ is the global optimal position, and ${X}_{worst}^{t}$ is the worst position that the discoverer is currently positioned at. $\beta$ is a parameter controlling the step size and has mean 0 and variance 1, obeying a normal distribution. $K$ is $\left.\left[-\text{1,1}\right.\right]$ the number of random numbers determining the direction of sparrow movement. ${f}_{i}$ is the current individual fitness value, ${f}_{g}$ is the current global optimal fitness value, ${f}_{w}$ is the current global worst fitness value, and $\varepsilon$ is the smallest constant that avoids a denominator of zero^26^. When ${f}_{i}>{f}_{g}$, sparrows are at the edge of the population, they are more vulnerable to predation. When ${f}_{i}={f}_{g}$, sparrows in the middle of the population realize the crisis and need to move closer to their peers to reduce the likelihood of predation.

SSA outperforms traditional optimization algorithms in terms of convergence speed, stability, and robustness^30^. However, the initial population of SSA is randomly initialized with great uncertainty, which has the disadvantages of low population diversity and making it easy to fall into local optimal solutions^31^. Therefore, the main improvement of SSA should focus on refining the population initialization^32^. The random generation of sparrows during the initialization stage makes the initial solution susceptible to aggregation, resulting in low coverage in the solution space and a low individual difference. However, the chaotic mapping initialization of the population can effectively address this issue. The circle mapping of chaos has the characteristics of randomness, uniformity, and order, which are more stable, and the coverage of the chaotic values is high. Therefore, in this paper, circle mapping is chosen to optimize the initial population to obtain the improved sparrow search algorithm (ISSA). The formula for generating the initial population by circle mapping is shown in Eq. (11):

11 $$\begin{array}{c}{x}_{n+1}=mod({x}_{n}+b-\left(\frac{a}{2\pi }\right)\text{sin}\left(2\pi {x}_{n}\right),1)\end{array}$$

where *n* is the dimension of the solution, *a* and *b* are the control parameters, ${x}_{n}$ is the chaotic solution, and *mod* denotes the residual.

## PMSM demagnetization fault diagnostic model

**Fig. 2:**
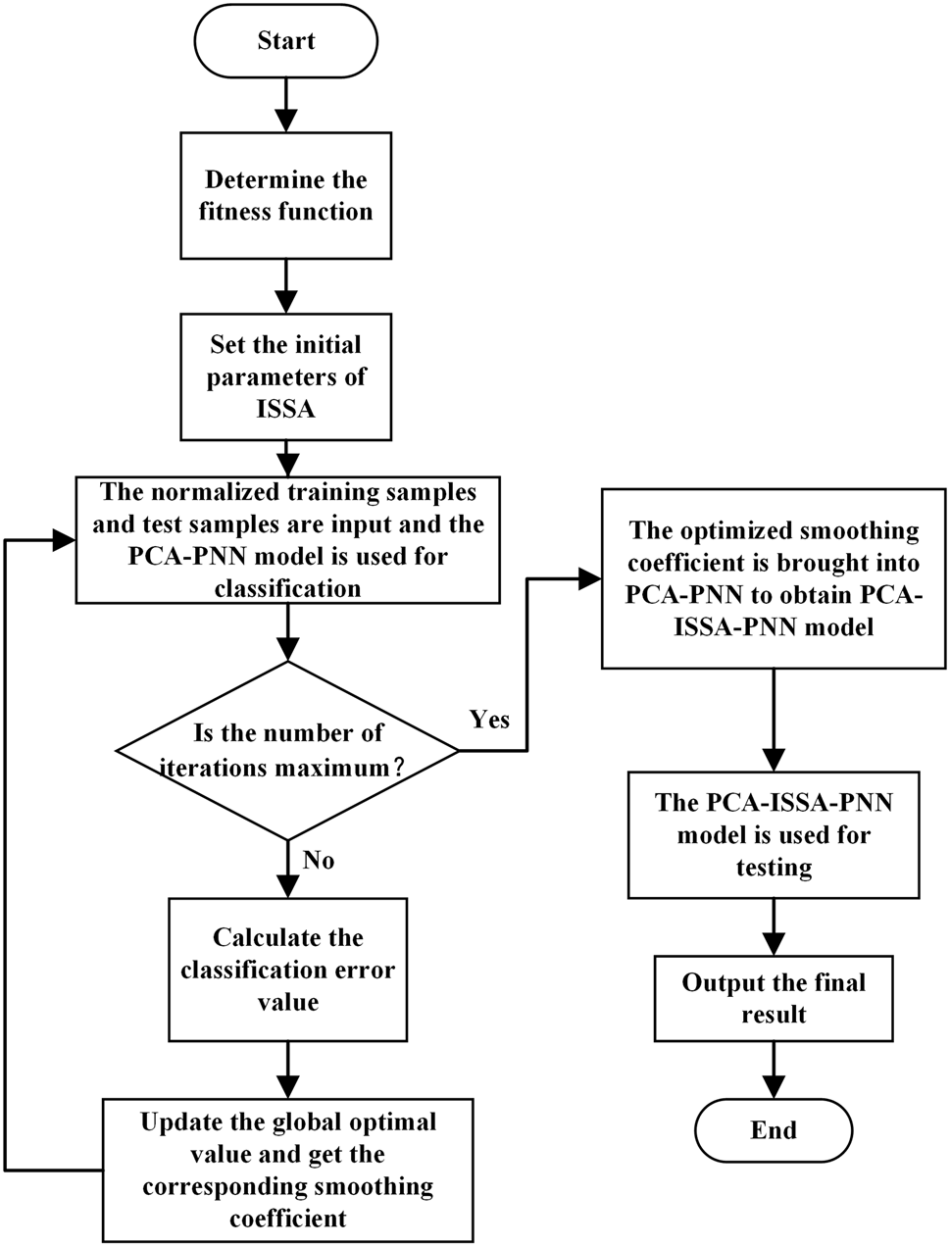
Flowchart of PCA-ISSA-PNN diagnosis model.

In this paper, we use the error value between the real result and the predicted result of PCA-PNN as the fitness function of ISSA, and the optimized parameters of ISSA as the smoothing coefficients of PCA-PNN. This allows us to combine the three algorithms of PCA, ISSA, and PNN, thereby improving the performance of fault diagnosis. The flowchart of the PCA-ISSA-PNN demagnetization fault diagnosis model is shown in Fig. 2. Firstly, the initial parameters of ISSA are set: the initial number of sparrow individuals is set to 10, the number of iterations is set to 30, and the proportion of the initial discoverer is set to 20% of the number of sparrow individuals. Then it inputs the normalized training samples and test samples, uses PCA-PNN for pattern recognition, calculates the classification error, and obtains the optimized parameter of ISSA through continuous iteration. Finally, the optimized parameter is brought into the smoothing coefficient to obtain the PCA-ISSA-PNN model for testing and validation.

## Experimental platform

**Fig. 3:**
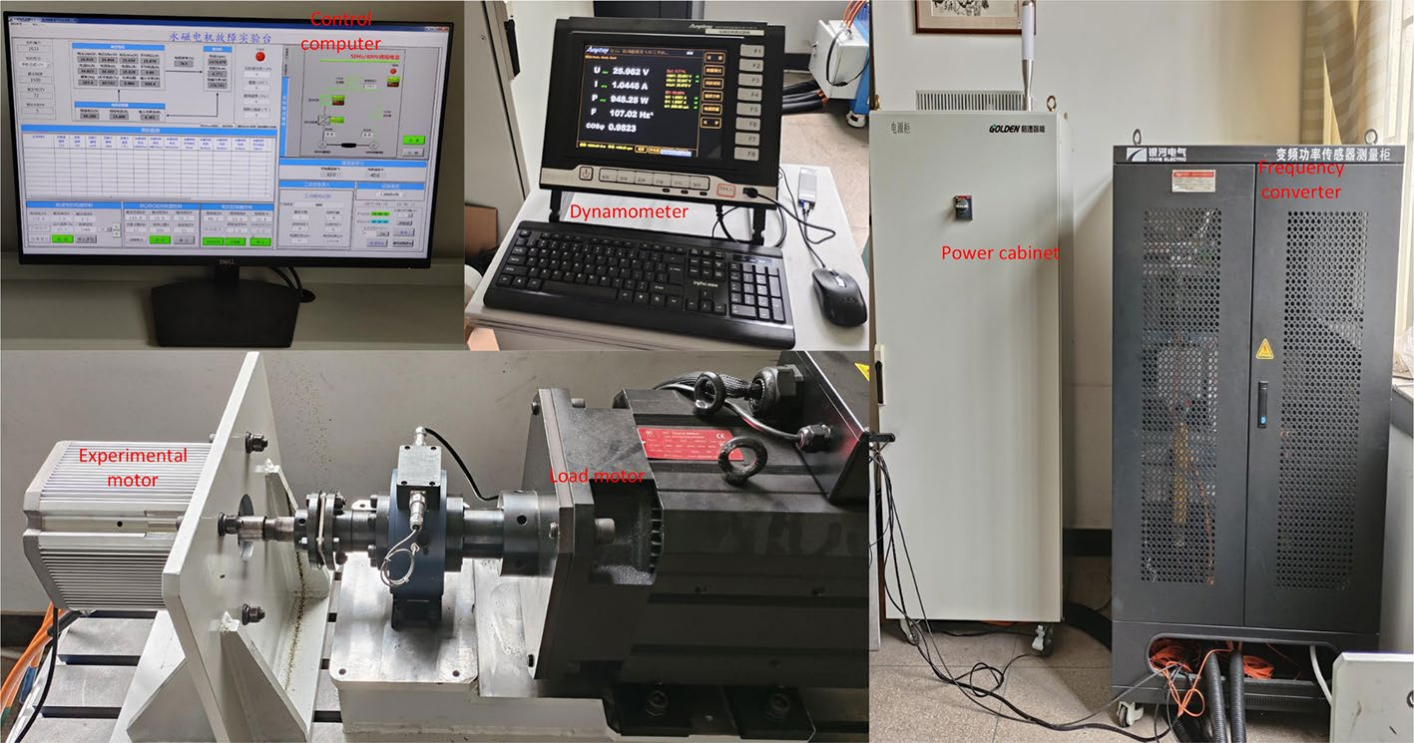
Permanent magnet synchronous motor fault experimental platform.

In order to verify the accuracy of the method in this paper and to obtain the PMSM normal and faulty phase current data, the experiment is carried out using the permanent magnet synchronous motor fault experimental platform, as shown in Fig. 3. The platform mainly includes an experimental motor, a load motor, a dynamometer, a power cabinet, a frequency converter, and a control computer. The experimental motors are normal motors with 50% unipolar demagnetization and 50% overall demagnetization, for a total of three motors. The structures are interior type. The related parameters of PMSM are shown in Table 1, and the related parameters of the load motor are shown in Table 2. Power cabinet input is three-phase 380-500 V voltage, frequency is 50 Hz, output is three-phase 380-500 V voltage, the frequency is 0-320 Hz, input and output current are 46A, rated power is 22 kW. The frequency converter model is SP332201C, the rated voltage is 3300 V, the rated current is 200A, the frequency test range is 0.1–1500 Hz, and the power is 1.5 kW. The function of the control computer is to control the platform to energize and de-energize, set the load torque, and motor speed.

**Table 1:** Related parameters of PMSM.

Model number	TZQ160-5-72
Rated power	5 kW
Rated voltage	72 V
Rated speed	3000 rpm
Peak speed	6800 rpm
Rated torque	19.9 $\text{N}\bullet \text{m}$
Number of pole pairs	4

**Table 2:** Related parameters of load motor.

Model number	DSM-38C015K-E0F3A24S
Rated power	15 kW
Rated voltage	380 V
Rated speed	1500 rpm
Peak speed	8000 rpm
Rated current	29.2 A
Reference frequency	50 Hz
Number of pole pairs	4

The data acquisition process involves the following steps: First, open the switch on the power cabinet and wait for its voltage to stabilize to 380 V. Next, the control computer activates the platform power supply; it then initiates the experimental and load motors, adjusting their rotational speeds to 500–3000 rpm and the load to a range of 1–10 N∙m. Finally, the WP4000 dynamometer, with a sampling frequency of 125 kHz, collects the data. It has a voltage measurement range of 100 μV–15 kV, a current measurement range of 100 μA–15 kA, and a measurement accuracy of 0.1%. We collect data once for every 100 rpm increase in rotational speed and every 1 N∙m increase in load torque.

## Experimental results and analysis

### Fault features extraction

**Fig. 4:**
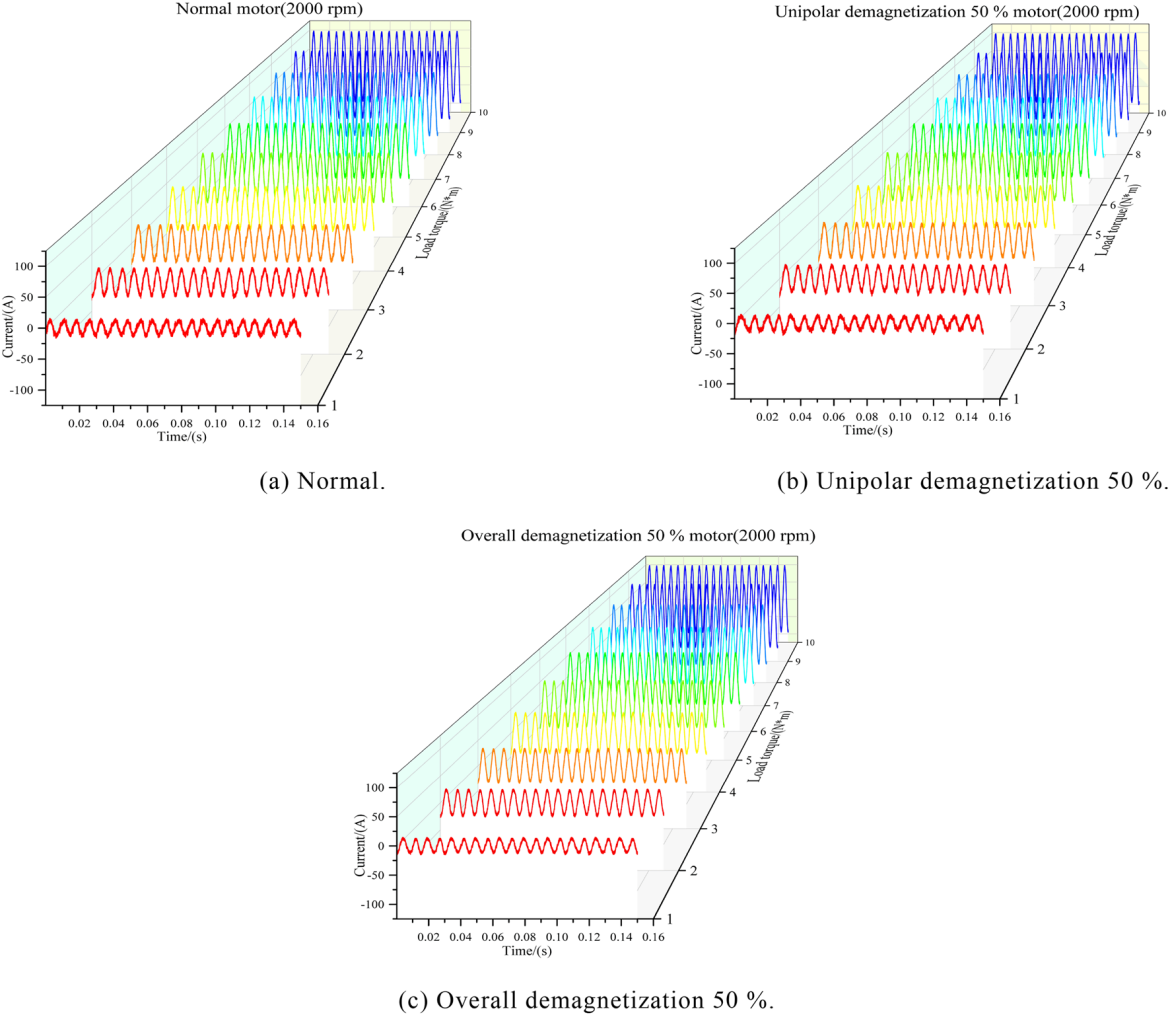
PMSM current-load waterfall diagram (2000 rpm).

**Fig. 5:**
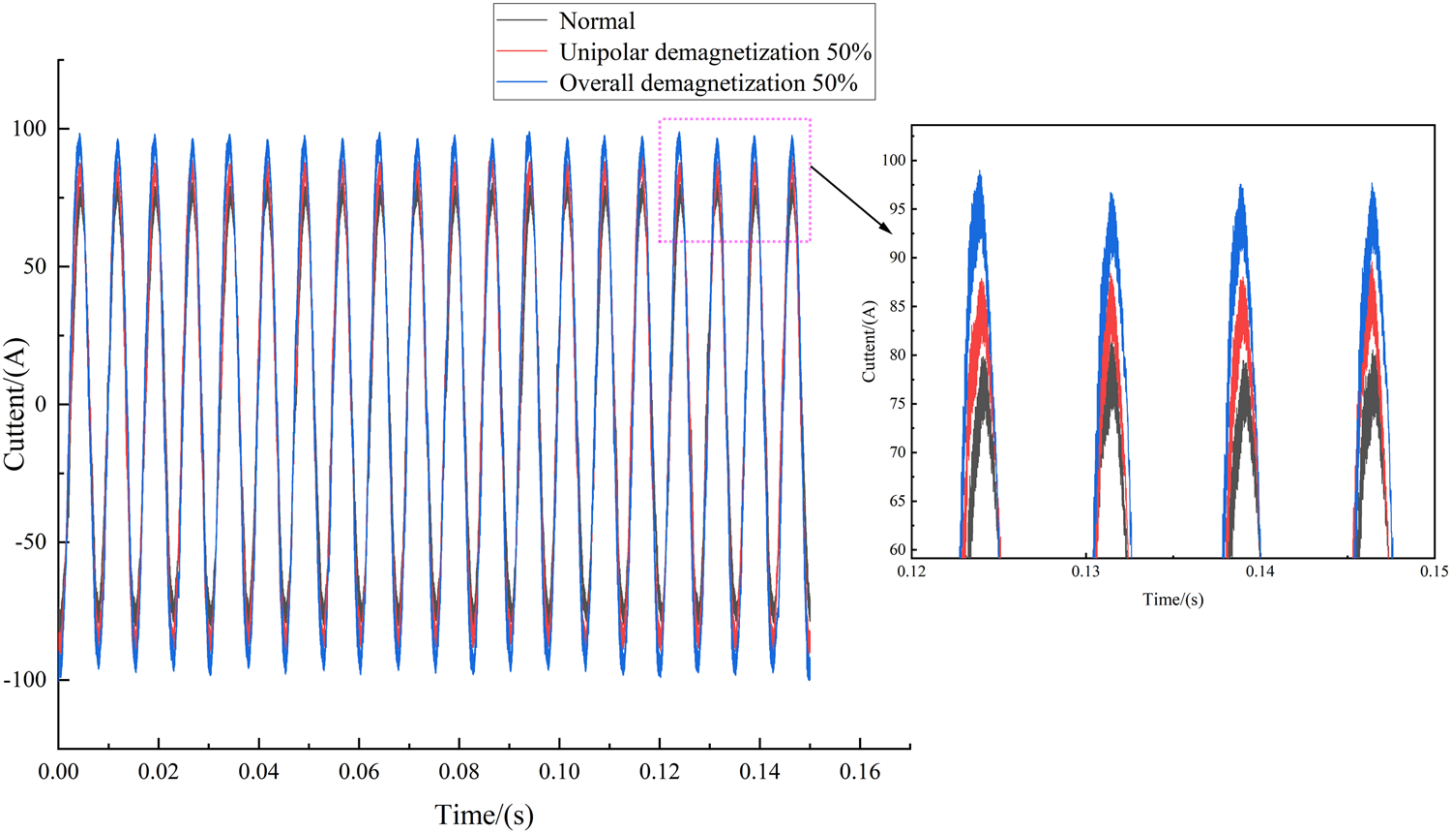
Current waveforms (9N $\cdot$ m: 2000 rpm).

The experiment selected 260 sets of current data under different working conditions: normal motor, 50% unipolar demagnetization, 50% overall demagnetization, motor speed range of 500–3000 rpm, load range of 1–10 nm, a total of 780 sets of data, each with an acquisition time of 0.15 s and 18,750 sample points. The current-load waterfall diagram of the PMSM for a speed of 2000 rpm and a load of 1–10 nm is shown in Fig. 4. The current waveforms of the three motors at a load of 9 Nm and a speed of 2000 rpm are shown in Fig. 5. From the figure, it can be seen that when the demagnetization fault occurs in the motor, the current will increase; the overall demagnetization 50% motor current amplitude is the largest; the unipolar demagnetization motor is the second largest; and the normal motor amplitude is the smallest.

**Fig. 6:**
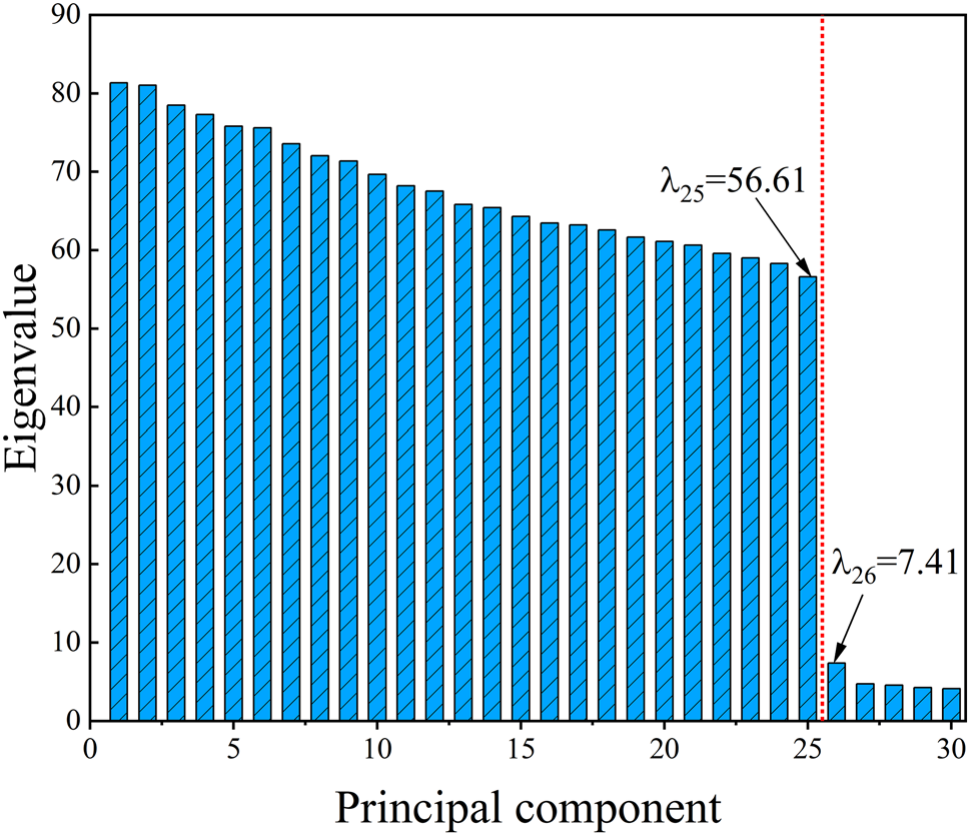
Eigenvalue ordering diagram.

**Fig.7:**
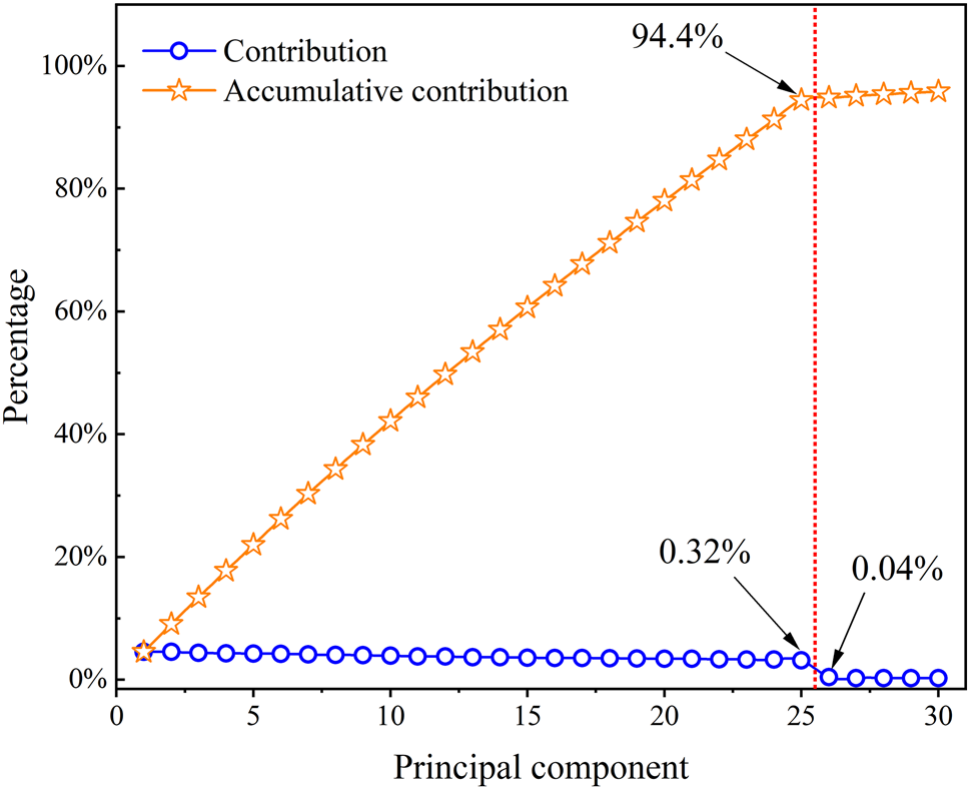
Contribution and accumulative contribution chart.

**Fig. 8:**
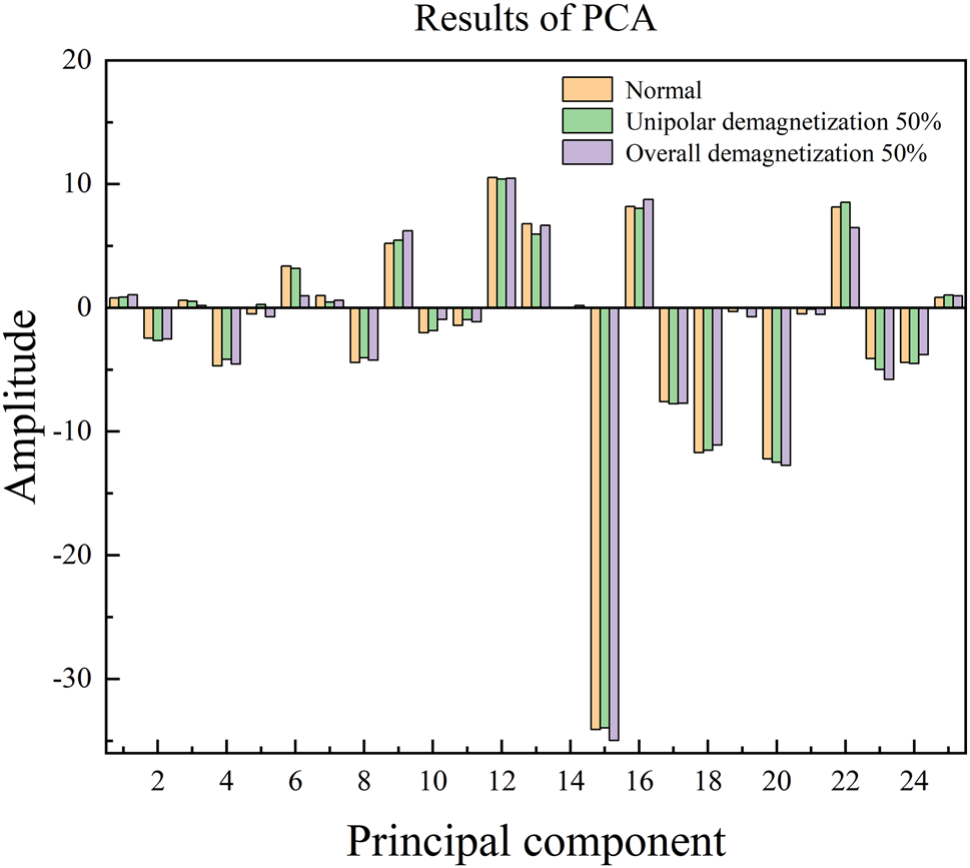
Results chart of PCA (9N $\cdot$ m: 2000 rpm).

To extract features using PCA, first create the initial matrix and calculate the correlation coefficient matrix. Second, calculate the eigenvalues corresponding to the correlation coefficient matrix and arrange them from largest to smallest, as shown in Fig. 6. Next, calculate the cumulative contribution and the contribution of individual principal components, as shown in Fig. 7. Figures 6 and 7 reveal that the subsequent principal components, after the 25th, have low eigenvalues and contribution degrees. Therefore, we can select the first 25 principal components to represent the majority of the data, with a corresponding cumulative contribution degree of 94.4%. Figure 8 shows the calculation of the last retained principal components.

### Experimental results analysis

In the experiment, 220 groups of normal motor, unipolar demagnetized 50% motor, and overall demagnetized 50% motor samples are selected as training samples, and the remaining 40 groups are used as test samples, with a total of 660 groups of training samples and a total of 120 groups of test samples, and the label designations are set to be 1, 2, and 3 in order. The pattern recognition is performed by using the PNN, PCA-PNN, PCA-GA-PNN, PCA-DA-PNN, PCA-GTO-PNN, PCA-AHA-PNN, PCA-SSA-PNN, and PCA-ISSA-PNN, respectively.

**Fig. 9:**
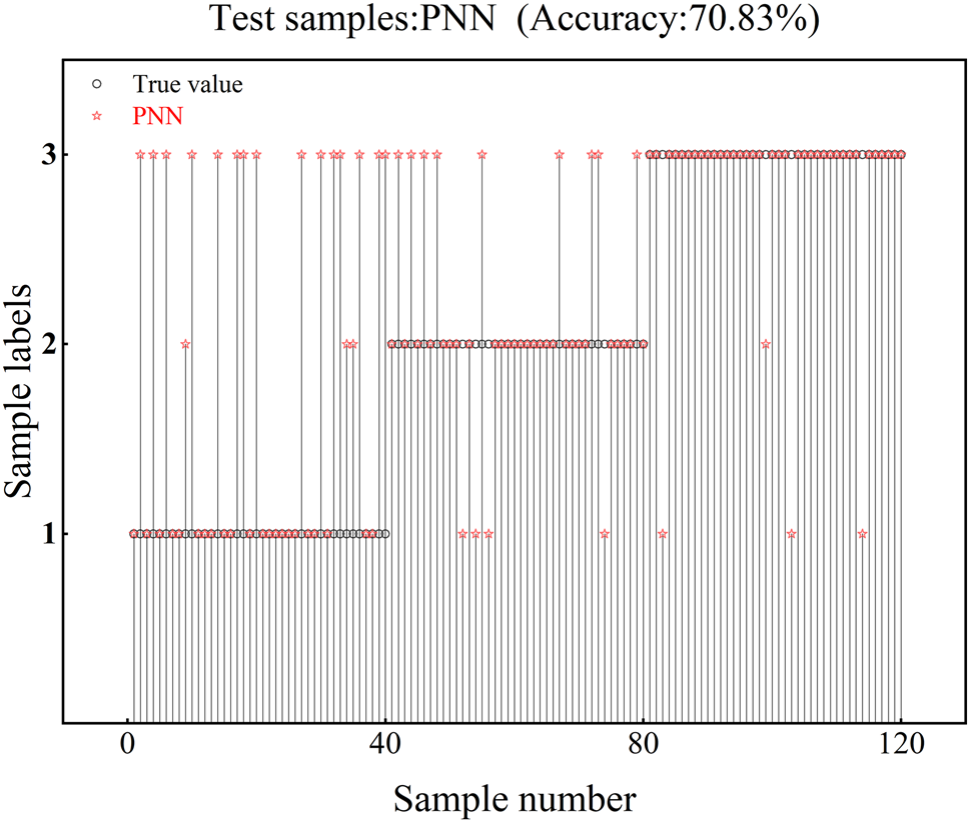
PNN verification results.

**Fig. 10:**
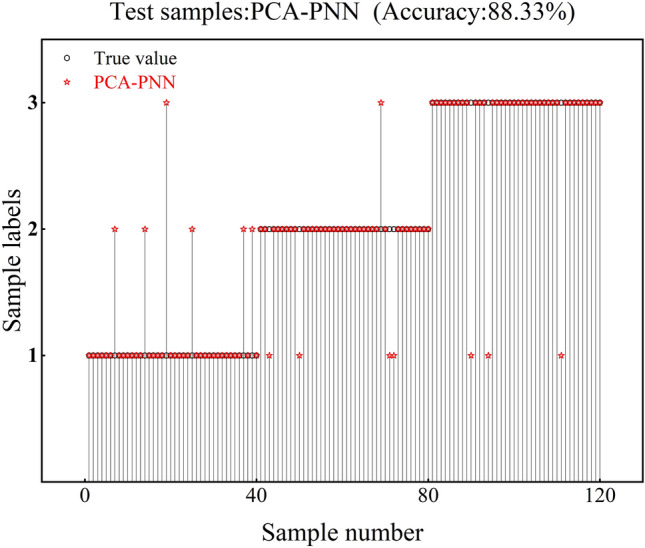
PCA-PNN verification results.

From the experimental results, it can be seen that the accuracy of the PNN test set is only 70.83%, and the test set has 18 recognition errors at class 1, 13 recognition errors at class 2, and 4 recognition errors at class 3, as shown in Fig. 9. The accuracy of the PCA-PNN test set was 88.33%, and the test set showed 6 recognition errors at class 1, 5 recognition errors at class 2, and 3 recognition errors at class 3, as shown in Fig. 10. The test set's accuracy is improved by 17.5% over PNN.

**Fig. 11:**
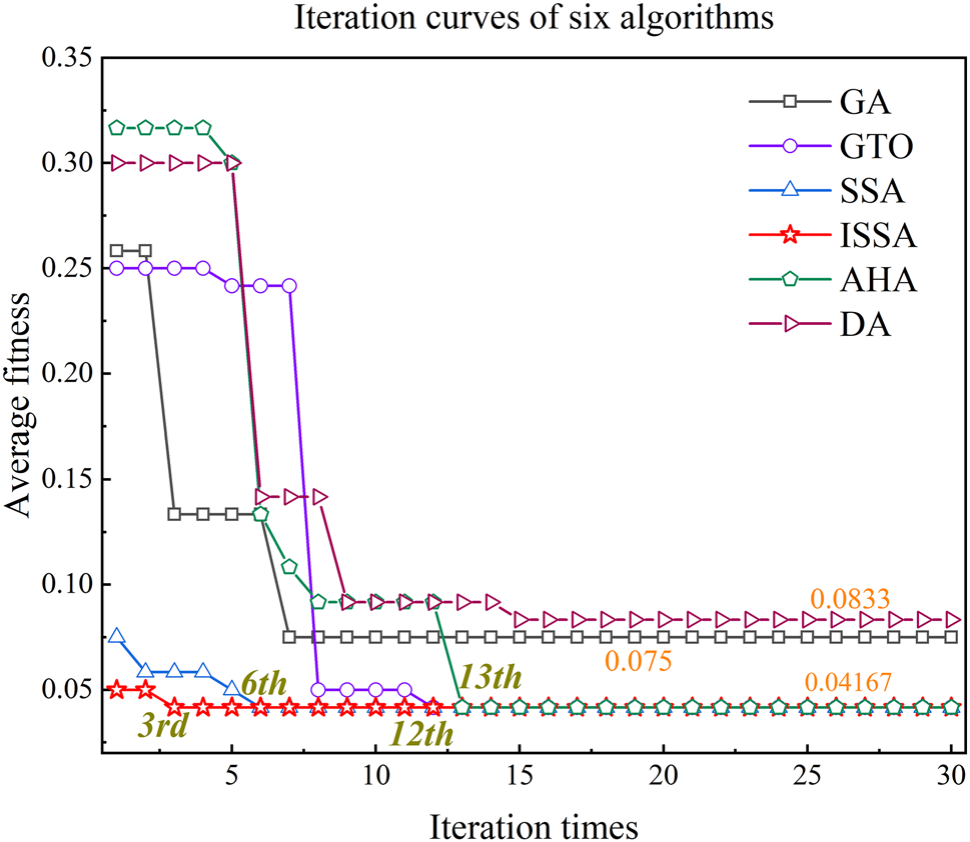
Iteration curves of six optimization algorithms.

The smoothing coefficient $\sigma$ optimization improved the accuracy of the PCA-GA-PNN, PCA-DA-PNN, PCA-GTO-PNN, PCA-AHA-PNN, PCA-SSA-PNN, and PCA-ISSA-PNN test sets. We ran the algorithms several times each, and obtained the average iteration curves of the six algorithms, as shown in Fig. 11. Figure 11 demonstrates that both GA and DA iterations achieve local optimality, resulting in corresponding values $\sigma$ of 0.7874 and 0.6456. GTO, AHA, SSA, and ISSA all reach the optimal solution, which is 0.089.

**Fig. 12:**
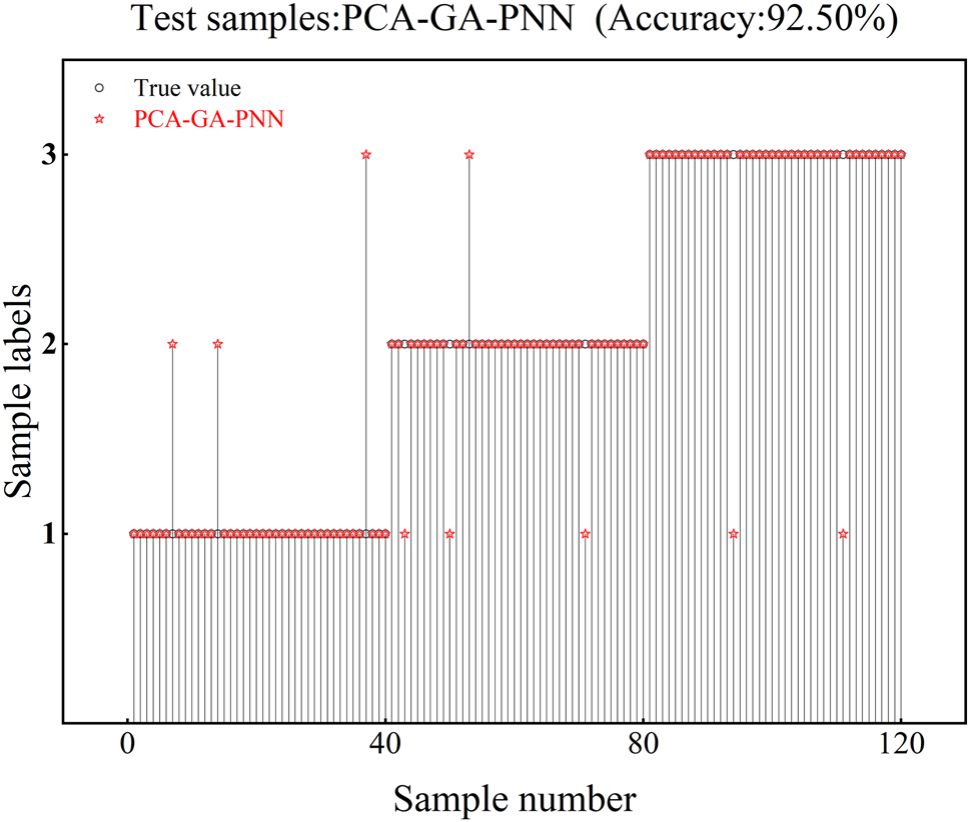
PCA-GA-PNN verification results.

**Fig. 13:**
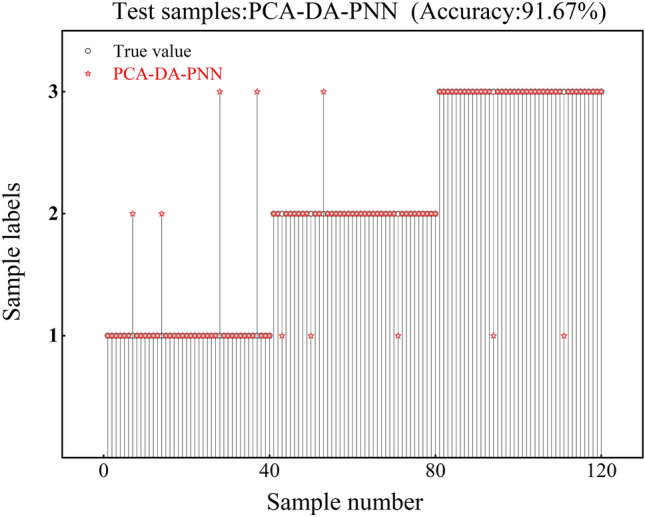
PCA-DA-PNN verification results.

**Fig. 14:**
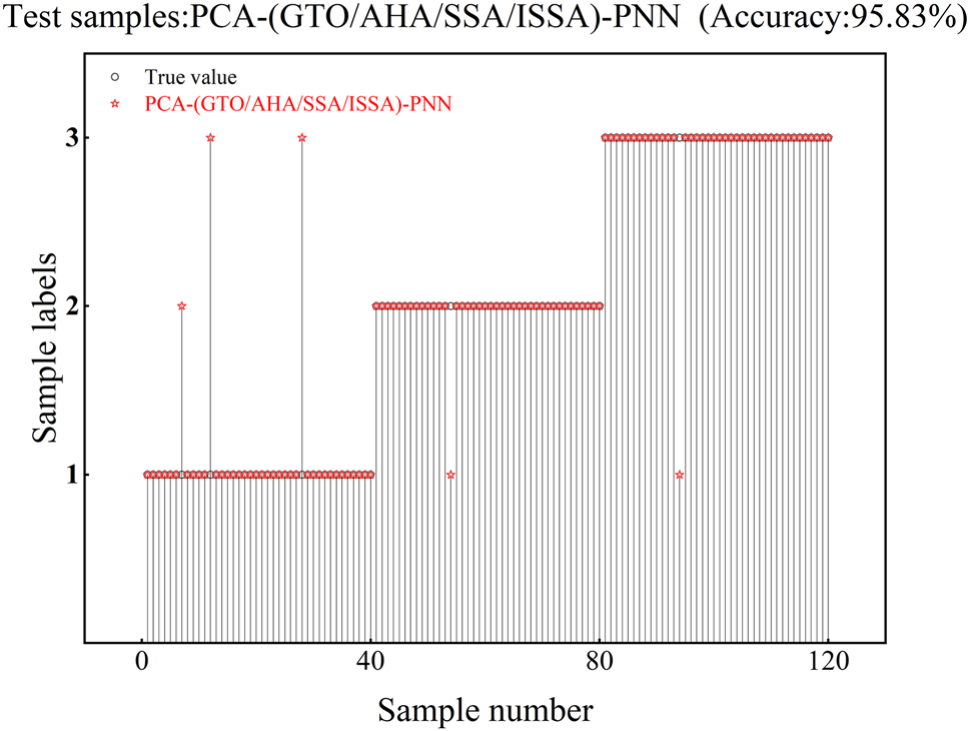
PCA-(GTO/AHA/SSA/ISSA)-PNN verification results.

The accuracy of the test set corresponding to PCA-GA-PNN is 92.5%, and the test set has 3 recognition errors in class 1, 4 recognition errors in class 2, and 2 recognition errors in class 3, as shown in Fig. 12. The accuracy of the test set corresponding to PCA-DA-PNN is 91.67%, and the test set has 4 recognition errors in class 1, 4 recognition errors in class 2, and 2 recognition errors in class 3, as shown in Fig. 13. The corresponding test set of PCA-(GTO/AHA/SSA/ISSA)-PNN has an accuracy of 95.83%, and it has 3 recognition errors in class 1, 1 recognition error in class 2, and 1 recognition error in class 3, as shown in Fig. 14.

The parameters related to the PNN network in the eight algorithms are shown in Table 3. From Table 3, it can be seen that all the PNN networks have a 4-layer structure with 660 nodes in layer 2, 3 nodes in layer 3 and 1 node in layer 4. However, the original PNN network has 18,750 nodes in layer 1. Due to dimensionality reduction using PCA, all other 7 algorithms have 25 nodes in layer 1. In this paper, only the smoothing coefficients $\sigma$ are optimized, so the values of the corresponding smoothing coefficients $\sigma$ are different after using the optimized algorithms, and the accuracy of the different algorithms will be different.

**Table 3:** Parameters associated with PNN networks in eight algorithms.

Algorithm	Layers	Layer 1 nodes	Layer 2 nodes	Layer 3 nodes	Layer 4 nodes	Smoothing coefficient $\sigma$
PNN	4	18,750	660	3	1	1.92
PCA-PNN		25				1.92
PCA-DA-PNN		25				0.6456
PCA-GA-PNN		25				0.7874
PCA-(GTO/AHA/SSA/**ISSA**)-PNN		25				0.089

**Fig. 15:**
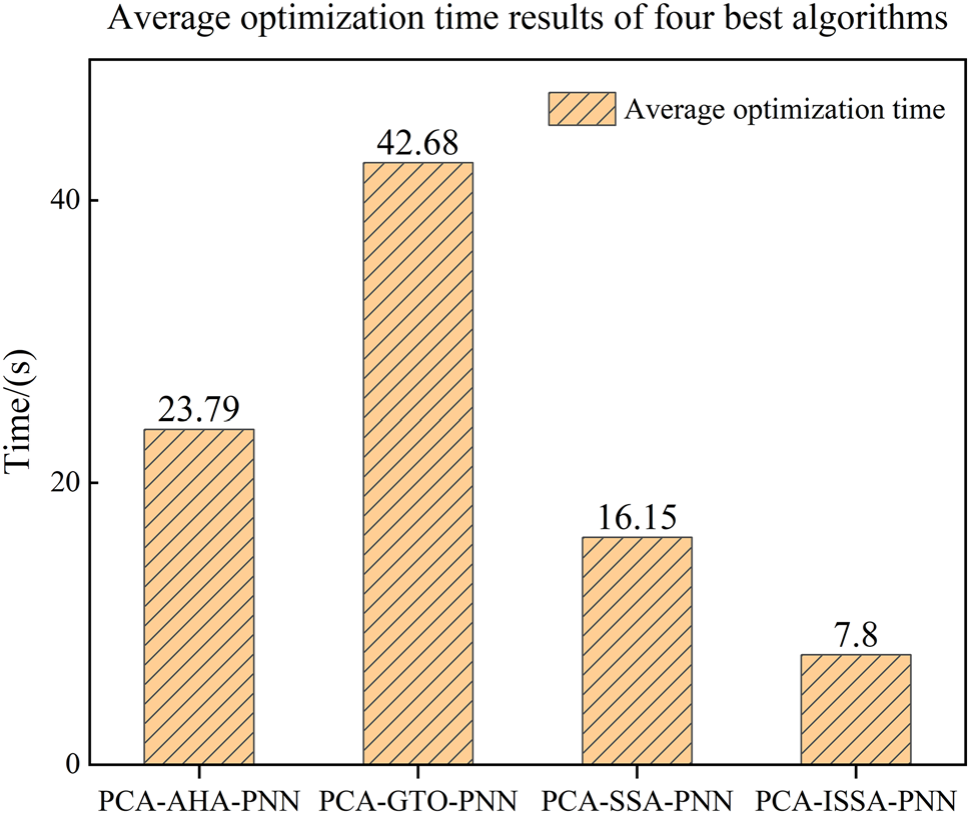
Performance comparison of four optimization algorithms.

The performance comparison of the six optimization algorithm models is shown in Table 4. From Table 4, it can be seen that GA and DA fall into the local optimum during optimization with average fitness of 0.075 and 0.0833, respectively, and GTO, AHA, SSA, and ISSA all reach the optimal solution with fitness of 0.4167. However, the average optimization time of ISSA is only 7.8 s, and the convergence takes place at the 3rd time on average, which is significantly lower than that of GTO, AHA, and SSA, as shown in Fig. 15, proving the high efficiency of ISSA.

**Table 4:** Performance comparison of six optimization algorithms.

Algorithm	Average optimization time	Average optimal fitness	Average number of iterations to optimization
PCA-GA-PNN	Not optimal	0.075	Not optimal
PCA-DA-PNN	Not optimal	0.0833	Not optimal
PCA-AHA-PNN	23.79s	0.4167	13th
PCA-GTO-PNN	42.68s	0.4167	12th
PCA-SSA-PNN	16.15s	0.4167	6th
**PCA-ISSA-PNN**	**7.8s**	**0.4167**	**3rd**

A comparison of the eight algorithms is shown in Table 5. From Table 5, it can be seen that PNN has the lowest recognition accuracy, precision per class, recall per class, and F1 score. When the PCA algorithm was added, the recognition accuracy reached over 88%, the precision rate for each class reached over 82%, the recall rate for each class reached over 85%, and the F1 scores reached over 83%, which demonstrated the high efficiency of PCA feature extraction. Among them, the PCA-ISSA-PNN accuracy reached 95.83%, the precision of each class reached more than 94.87%, the recall of each class reached more than 92.5%, and the F1 scores reached more than 93.67%, which were significantly higher than the other models. It proves that the PCA-ISSA-PNN model has good fault diagnosis indexes and can identify demagnetization faults in motors with high precision and efficiency.

**Table 5:** Comparison results of eight algorithms.

Algorithm	Accuracy (%)	Precision (%)			Recall (%)			F1 score (%)		
		1	2	3	1	2	3	1	2	3
PNN	70.83	75.86	87.1	60	55	67.5	90	63.77	76.06	72
PCA-PNN	88.33	82.93	87.5	94.8	85	87.5	92.5	83.95	87.5	93.64
PCA-DA-PNN	91.67	87.8	94.74	92.68	90	90	95	88.89	92.32	93.83
PCA-GA-PNN	92.5	88.1	94.74	95	92.5	90	95	90.54	92.32	95
PCA-(GTO/AHA/SSA/**ISSA**)-PNN	**95.83**	**94.87**	**97.5**	**95.12**	**92.5**	**97.5**	**97.5**	**93.67**	**97.5**	**96.30**

The experimental dataset used in this paper is only a segment of the real dataset selected for experiments and dimensionality reduction, so the sample size of the experimental dataset is smaller compared to the real dataset, which may have the following effects on the model.

1. The sample dimension of PCA after dimensionality reduction is smaller compared with the real dataset, which may lose some information, thus affecting the accuracy and recall of the model.
2. The number of samples and the length of samples selected for the experiment may not fully reflect all the information in the real dataset, so the model trained on the experimental dataset may not generalize well to the real dataset.
3. Real datasets are large and complex, and using real datasets for training and evaluation may require more computational resources and time. Experimental datasets, on the other hand, can reduce these costs to some extent.

## Conclusion

This paper proposes a PMSM demagnetization fault diagnosis method based on PCA-ISSA-PNN, using the interior PMSM as the research object. The established fault diagnosis model is tested and validated using the current data collected from the experiment. The validation results show that:

1. The accuracy of the PMSM fault diagnosis model based on PCA-ISSA-PNN reaches 95.83%, which is higher than the fault diagnosis accuracies of 70.83% for the PNN network, 88.33% for the PCA-PNN network model, 92.5% for the PCA-GA-PNN network model, and 91.67% for the PCA-DA-PNN network model. The accuracy of the PCA-ISSA-PNN class has higher accuracy, recall, and F1 score than them, and its fault diagnosis index is clearly optimal.
2. The PCA-ISSA-PNN-based PMSM fault diagnosis model has a significantly lower average running time and average number of convergences than PCA-GTO-PNN, PCA-AHA-PNN, and PCA-SSA-PNN. PCA-ISSA-PNN has better fault diagnosis performance.

The accuracy and efficiency of the proposed PCA-ISSA-PNN-based fault diagnosis method are demonstrated. The method solves the problem of unsatisfactory diagnostic effects due to the artificial setting of smoothing coefficients in the PNN network, improves the accuracy of PMSM demagnetization fault diagnosis, and has good prospects for popularization and application.

### Future work

In this paper, we only improve the initial population of SSA, and in the future, we will improve the optimization strategy of SSA to further optimize it. Due to the limitations of experimental conditions, the method proposed in this paper is only applied to three kinds of fault classification at present, and in the future, we will try to diagnose the multiple kinds of faults in PMSM by using this method. Additionally, readers can apply this method to other fields and experiment with various feature extraction methods, which could potentially lead to improved classification results.

## Data Availability

The datasets used and analysed during the current study available from the corresponding author on reasonable request.
